# Insecure attachment during infancy predicts greater amygdala volumes in early adulthood

**DOI:** 10.1111/jcpp.12317

**Published:** 2014-08-23

**Authors:** Christina Moutsiana, Tom Johnstone, Lynne Murray, Pasco Fearon, Peter J Cooper, Christos Pliatsikas, Ian Goodyer, Sarah L Halligan

**Affiliations:** 1 Division of Psychology and Language Sciences, University College LondonLondon, UK; 2 School of Psychology and CLS, University of ReadingReading, UK; 3 Department of Psychology, Stellenbosch UniversityStellenbosch, UK; 4 School of Psychology, University of KentCanterbury, UK; 5 Department of Psychiatry, University of CambridgeCambridge, UK; 6 Department of Psychology, University of BathBath, UK

**Keywords:** Attachment, brain development, amygdala, longitudinal, maternal depression

## Abstract

**Background:**

The quality of the early environment is hypothesized to be an influence on morphological development in key neural areas related to affective responding, but direct evidence to support this possibility is limited. In a 22-year longitudinal study, we examined hippocampal and amygdala volumes in adulthood in relation to early infant attachment status, an important indicator of the quality of the early caregiving environment.

**Methods:**

Participants (*N* = 59) were derived from a prospective longitudinal study of the impact of maternal postnatal depression on child development. Infant attachment status (24 Secure; 35 Insecure) was observed at 18 months of age, and MRI assessments were completed at 22 years.

**Results:**

In line with hypotheses, insecure versus secure infant attachment status was associated with larger amygdala volumes in young adults, an effect that was not accounted for by maternal depression history. We did not find early infant attachment status to predict hippocampal volumes.

**Conclusions:**

Common variations in the quality of early environment are associated with gross alterations in amygdala morphology in the adult brain. Further research is required to establish the neural changes that underpin the volumetric differences reported here, and any functional implications.

## Introduction

Animal research has established that early stress exposure is a key influence on neural development in regions that are central to affective responding and stress reactivity (Huang & Lin, 2006; Meaney, 2001; Ono et al., 2008; Raineki, Cortes, Belnoue, & Sullivan, 2012). In particular, rodent studies have demonstrated that maternal separation or poor care may result in decreased neurogenesis and glucocorticoid receptor density in the hippocampus (Huot, Plotsky, Lenox, & McNamara, 2002; Ladd, Huot, & Plotsky, 2000), with associated hippocampal volumetric reductions (Meaney, 2001). Early stress exposure has also been linked to altered amygdala morphology and function in animal studies (Caldji et al., 1998); disrupted maternal care in rodents has been associated with precocious myelination (Ono et al., 2008) and enhanced amygdala activation in response to stress (Huang & Lin, 2006; Raineki et al., 2012). These observations are of considerable interest, given the central role that these regions play in emotional processing and responding. The amygdala is a major component of the neural circuitry of emotion, contributing to detection and evaluation of emotionally salient stimuli, as well as the expression of emotional responses such as fear (Whalen & Phelps, 2010). By contrast, the hippocampus exerts strong regulatory control over the hypothalamic–pituitary–adrenal (HPA) axis stress response system, ultimately serving to modulate cortisol output via hippocampal glucocorticoid receptors (Dedovic, Duchesne, Andrews, Engert, & Pruessner, 2009). Although the findings from nonhuman primate studies concerning longer term effects of maternal separation on brain development are more complicated, such observations from the animal literature have stimulated investigation of whether human neural development may be similarly sensitive to the quality of care received in early life (Gunnar & Quevedo, 2007).

Early experience has been examined in relation to human hippocampal and amygdala morphology across several contexts, with some consistent observations emerging. Adults with PTSD who experienced maltreatment in childhood show smaller hippocampi than their nonmaltreated counterparts, although an equivalent effect has not been found in children (McCrory, De Brito, & Viding, 2010). In addition, in a study of normative variations in maternal care, higher levels of maternal support in early childhood were found to be strongly predictive of larger hippocampal volumes in school-age children (Luby et al., 2012). With regard to the amygdala, overt childhood maltreatment has not been linked to morphology, at least as evidenced by the gross alterations indexed by volumetric measurement (McCrory et al., 2010). However, enlarged amygdala volumes have been observed in two samples of orphanage-reared children, where environments are characterized by neglect (Mehta et al., 2009; Tottenham et al., 2010). In addition, Lupien and colleagues found amygdala volumes to be increased in the context of chronic maternal depressive symptoms, an association that was hypothesized to be a consequence of the withdrawn parenting that typically occurs in this context (Lupien et al., 2011). Direct observation of aspects of the parent–child relationship is required to confirm that variations in the early nurturing environment are linked to gross alternations in amygdala morphology.

The attachment bond between an infant and their primary caregiver is considered to be a biologically primed, fundamental aspect of the early caregiving environment (Ainsworth, Blehar, Waters, & Wall, 1978; Bowlby, 1979). Securely attached infants experience interactions with their primary caregivers that are broadly sensitive and responsive to the infant's emotional needs, whereas insecurely attached infants are more often subject to inconsistent, withdrawn or negative/rejecting caregiver responses (Ainsworth et al., 1978; de Wolff & van IJzendoorn, 1997). Correspondingly, different patterns of infant attachment behaviours are displayed. Infants with secure attachment patterns tend to seek proximity when anxious and find contacting effective in reducing their distress. Insecure attachment patterns, where infants may avoid contact, become angry and inconsolable or disorganized in their attachment behaviours, are thought to represent suboptimal behavioural strategies for responding to threat which leave insecurely attached infants more vulnerable to stress than their secure counterparts (Cassidy & Shaver, 2008; Fox & Hane, 2008). Attachment is thus a potentially important mechanism via which the quality of care may influence the developing child's experience of and responding to stress. Consistent with this, several studies have indicated that children who show insecure attachment patterns respond with heightened cortisol responses to separation and to other stressors relative to secure children (Ahnert, Gunnar, Lamb, & Barthel, 2004; Hertsgaard, Gunnar, Erickson, & Nachmias, 1995; Spangler & Grossmann, 1993).

Early experiences of stress that occur in association with attachment insecurity may, in principle, serve to influence neural development in sensitive regions including the hippocampus and amygdala (Gunnar & Quevedo, 2007; Lupien, McEwen, Gunnar, & Heim, 2009). Little direct evidence on this point is available. However, previous work by our group supports the possibility of altered neural outcomes in relation to infant attachment security; in a functional magnetic resonance imaging (fMRI) study, we found evidence of less effective prefrontal neural engagement during attempts to upregulate positive emotions in young adults who were insecurely versus securely attached infants (Moutsiana et al., 2014). Here, in the same longitudinally studied sample, we investigated whether insecure versus secure early mother–infant attachments predict hippocampal and/or amygdala volumes in adulthood, as a preliminary test of the hypothesis that key aspects of the caregiving environment that are captured by attachment status may influence morphological neurodevelopment. Our sample, originally recruited shortly following birth, comprised individuals whose mothers were suffering from postnatal depression (PND; *n* = 28) together with control participants whose mothers did not have PND (*n* = 31). Attachment status was assessed at 18 months via direct observation using the Strange Situation, the gold standard in the field; and structural MRI scans were completed at age 22 years. We hypothesized that insecure versus secure attachments would be associated with smaller hippocampal volumes, and/or larger amygdalae.

Several covariates were considered. First, whole brain volume, hemisphere/laterality and gender were taken account of in all analyses, as each of these typically explains important variance in neural structures (Brain Development Cooperative Group, 2011; Free et al., 1995). We also considered maternal depression and participant's own history of depression and anxiety as potential covariates for analyses. Previous research has linked the presence of youth or adult depression (Caetano et al., 2007; Campbell, Marriott, Nahmias, & MacQueen, 2004; MacMaster et al., 2008; Videbech & Ravnkilde, 2004), and also family history of depression (Baare et al., 2010; Chen, Hamilton, & Gotlib, 2010; Rao, Chen, et al., 2010) with reduced hippocampal volumes, although not always consistently (Luby et al., 2012; Lupien et al., 2011; Pannekoek et al., 2014; Rosso et al., 2005). Similarly, there have been observations of enlarged amygdalae in association with familial risk of depression (Lupien et al., 2011; Romanczuk-Seiferth et al., 2014), and with individual symptoms of anxiety (Baur, Hanggi, & Jancke, 2012; MacMillan et al., 2003; Qin et al., 2014; Tottenham et al., 2010), although again there have been some mixed findings (Munn et al., 2007). Given that maternal PND was prevalent in our sample and that significant rates of anxiety and depressive disorders were also identified in our participants by adolescence (Halligan, Murray, Martins, & Cooper, 2007), these factors were considered to be important possible confounds.

## Methods

Procedures were approved by the University of Reading and the National Health Service Research Ethics Committees. Participants provided written informed consent prior to taking part.

### Participants

Participants were derived from a prospective longitudinal study of the development of children of postnatally depressed and well women (Murray, 1992). The sample was originally recruited at 2 months postpartum, through screening a community sample of primiparous mothers of healthy, full-term infants for PND. To be considered for inclusion in the study, mothers needed to be aged 18 years or older, married or cohabiting, and the primary caregiver for the infant. The Edinburgh Postnatal Depression Scale (EPDS; Cox, Holden, & Sagovsky, 1987) was administered at 6 weeks postpartum, and women scoring over 12 were interviewed; 61 women who met research diagnostic criteria (Spitzer, Endicott, & Robins, 1978) for depressive disorder were identified, 58 of whom were recruited for the study. Forty-two nondepressed mothers were also recruited from the same postnatal population; once a PND group mother was recruited, the next mother to screen negative for PND was invited to take part in this control group. All control group mothers identified agreed to participate. (For full details of recruitment see Murray, 1992).

At 22 years of age, 45 (76%) offspring from the PND group and 39 (93%) from the comparison group were available for the study. Of these, 63 (i.e. 75% of the available sample) were able to complete MRI scanning (exclusions were due to diabetes, epilepsy, metal implants, and inability to attend a session at the University). Complete structural scans were not obtained for one participant, and a further three participants were excluded due to structural abnormalities (*n* = 2) or inadequate image resolution (*n* = 1). The final sample comprised 29 females and 30 males, mean age 22 years.

### Measures

#### Infant attachment

At 18 months infant attachment was assessed using the Ainsworth's Strange Situation Procedure, the gold standard observational measure of attachment security (Ainsworth et al., 1978; Main & Solomon, 1990). Attachment patterns are categorized according to the infant's response to separation from, and particularly reunion with, a primary caregiver (in this case the mother) in an unfamiliar environment. The assessment yields four classifications: a ‘secure’ category, and three nonsecure categories (insecure avoidant, insecure ambivalent, and disorganized). Securely attached infants show a positive response to being reunited with their parent, and, if distressed by the separation, are easily comforted. Insecure avoidant infants show little distress at separation and limited proximity seeking and/or active avoidance of the parent upon reunion. Insecure ambivalent infants show high levels of distress at separation and proximity seeking upon reunion, but cannot be readily consoled by their parent. Finally, infant disorganization/disorientation is characterized by a range of incoherent or contradictory behaviours during separations and reunions or signs of fear in relation to the parent. Attachment was classified from videotapes following the standard procedures (Ainsworth et al., 1978; Main & Solomon, 1990). Cohen's kappa coefficient for the secure, avoidant, ambivalent, and disorganized attachment classifications of the two researchers who independently scored 63 randomly selected videotapes of the Strange Situation was.94. Following the majority of previous studies, we based analyses on comparisons of secure versus insecure infants.

#### Offspring psychological disorder

Offspring depressive and anxiety disorders were indexed via the Structural Clinical Interview for DSM-IV at 22 years (Spitzer, Williams, & Gibbon, 1995); and at 8, 13 and 16 years via the Kiddie Schedule for Affective Disorders and Schizophrenia, Present and Lifetime Version (Kaufman et al., 1997). At 22 years alcohol and substance abuse/dependence were also assessed. Interviews were conducted by trained researchers blind to group status. At every time point, participants reported on current and previous disorder, and the resultant information was used to establish lifetime diagnoses (present/absent).

#### Maternal depression

Maternal depression was assessed using the SCID-IV at 22 years by interviewers blind to group. Clinical interviews were similarly conducted at 18 month, 5 year, 8 year, 13 year and 16 year assessments, meaning that we were able to establish total number of months of maternal depression over the offspring's lifetime.

#### MRI data acquisition and processing

High-resolution three-dimensional (3D) T1-weighted images were acquired on a 3-T whole-body scanner (Siemens MAGNETOM Trio) with a 12-channel Head Matrix coil. The MRI parameters of the 3D magnetization-prepared rapid gradient-echo (3D-MPRAGE sequence) were the following: FOV = 250 × 250 mm^2^, TR/TE/TI/FA = 2020 ms/2.52 ms/1.1/9°. The images were acquired with an in-plane spatial resolution of 0.9765 mm and with 176 contiguous sagittal 1-mm-thick slices. Thus, nearly isotropic three-dimensional MR data sets were obtained, to allow accurate volumetric MR measurements.

Raw structural data were converted from the DICOM format into the NIfTI format through the dcm2nii software (Rorden & Brett, 2000). We first inspected all raw images for abnormalities. Images from three participants were excluded from the analysis, two of them due to structural abnormalities, and one due to very poor resolution because of head motion. All data processing was carried out using FSL version 4.1.8 (FMRIB's Software Library: http://www.fmrib.ox.ac.uk/fsl; Smith et al., 2004; Woolrich et al., 2009). Nonbrain tissue was removed from the high-resolution anatomical images using BET (Smith, 2002), and the remaining voxels were summed up to give an estimate of the total intracranial volume (TIV) per participant, which we used as a covariate in subsequent analysis. Volume of interest analysis was carried out with individual hippocampal and amygdala masks using the automated FSL tool, FIRST version 1.2 (FMRIB's Integrated Registration and Segmentation Tool). FIRST is widely used for volumetric analyses of subcortical brain units, with results comparable with those obtained through semiautomated manual segmentation; and yields volumetric estimates on bilateral amygdalae and hippocampal units that are highly comparable to manual tracing (Morey et al., 2009). Nonzero voxels/volumes within the masks were calculated in mm^3^ using Fslstats (http://fsl.fmrib.ox.ac.uk/fsl/fslwiki/Fslutils). All volumetric analyses were performed in subject-native space (i.e. not in MNI template space).

## Results

Sample characteristics are presented in Table1, by attachment status; age was highly constrained (range 21–23 years), and rate of psychotropic medication use was extremely low. As previously described for this sample, nonsecure attachments were almost exclusively of the avoidant subtype (*n* = 21) versus ambivalent (*n* = 1) or disorganized (*n* = 2); and the PND versus control group contained a higher proportion of participants who were insecurely attached as infants (Murray, Halligan, Adams, Patterson, & Goodyer, 2006). Similarly, there was a trend for total months of maternal depression during the course of the study to be higher for insecure versus secure participants.

**Table 1:** Sample characteristics reported by attachment status

	18-month attachment status	
	Secure (*N* = 35)	Insecure (*N* = 24)
Age in years, *M* (*SD*)	22.3 (.65)	22.5 (.64)
Proportion female, *N* (%)	20 (57.1)	9 (37.5)
Social class I, II and III nonmanual, *N* (%)	33 (71.7)	19 (61.3)
Education after 18 years, *N* (%)	27 (61.4)	22 (75.9)
Lifetime depressive disorder, *N* (%)	8 (22.9)	10 (41.7)
Lifetime anxiety disorder, *N* (%)	9 (25.7)	7 (29.2)
Any psychotropic medication use, *N* (%)	2 (6.9)	1 (3.3)
Maternal postnatal depression, *N* (%)	12 (34.3)	16 (66.7)**
Total months maternal depression, *M* (*SD*)	16.2 (24.8)	29.5 (29.8)*

* ^*^p <* *.10;

* ^*^^*^p <* *.001; PND, postnatal depression.

Key sample characteristics, namely maternal PND status, total months of maternal depression, and participant's own history of depression or anxiety (present/absent), level of education and socioeconomic status, were screened as potential covariates by examining their relationship with volumetric outcomes. Multiple linear regression analyses were conducted, examining total hippocampal/amygdala volumes in relation to each potential confound, controlling for TIV and gender. Results are presented in Table2. For hippocampal volume, significant positive associations were observed for maternal total months of depression and the presence of a lifetime history of depressive disorder in the participant, but there were no other significant effects. There was no evidence that amygdala volume was related to any of the covariates. Based on these observations, maternal total study months of depression and participant history of depression were controlled for in analyses of hippocampal volume. Gender and TIV were included as covariates for all analyses.

**Table 2:** Results of linear regression analyses examining hippocampal and amygdala volume in relation to maternal postnatal depression, total study months of depression, participant history of depression and anxiety, education and socioeconomic status; separate analyses conducted for each variable

	Total hippocampal volume		Total amygdala volume	
	Parameter	Model^a^	Parameter	Model^b^
Maternal postnatal depression^c^	*β *= .10, *p *=* *.39	*R*^2^* = *.26, *p *=* *.001	*β *= .01, *p *=* *.93	*R*^2^* = *.07, *p *=* *.25
Maternal total months depression	*β *= .24, *p *=* *.038	*R*^2^* = *.32, *p < *.001	*β *= .12, *p *=* *.38	*R*^2^* = *.08, *p = *.22
Participant lifetime depressive disorder^c^	*β *= .23, *p *=* *.046	*R*^2^* = *.30, *p *<* *.001	*β *= .10, *p *=* *.46	*R*^2^* = *.08, *p *=* *.30
Participant lifetime anxiety disorder^c^	*β *= .12, *p = *.34	*R*^2^* *=* *.26, *p *=* *.001	*β *= −.04, *p *=* *.77	*R*^2^* *=* *.07, *p = *.25
Education^d^	*β *= .16, *p *=* *.18	*R*^2^* *=* *.29, *p *=* *.001	*β = *−.02, *p = *.86	*R*^2^* *=* *.07*, p = *.29
Socioeconomic status	*β *= −.17, *p *=* *.15	*R*^2^* *=* *.27, *p *<* *.001	*β *= .05, *p *=* *.69	*R*^2^* = *.07, *p = *.24

a Model includes total intracranial volume and gender, *R*^2^* = *.25, *F*_2,56 _= 9.27, *p *<* *.001.

b Model includes total intracranial volume and gender, *R*^2^* = *.07, *F*_2,56 _= 2.15, *p *=* *.13.

c 0 = disorder absent, 1 = disorder present.

d 1 = age 16 years educational qualification equivalent, 2 = age 18 years equivalent, 3 = more than 18 years equivalent.

### Infant attachment at 18 months

**Figure 1:**
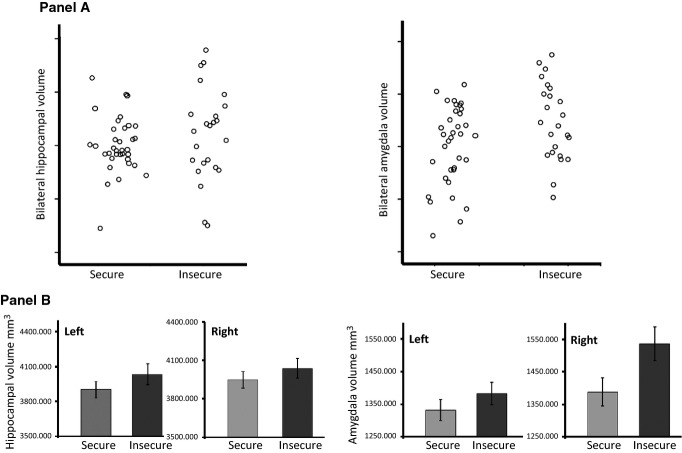
Hippocampal and Amygdala volumes in the secure and insecure attachment groups. Panel A: bilateral volumes are plotted with correction for total intracranial volume using regression analysis; actual values are regression residuals. Panel B: estimated marginal means (with standard errors) for left and right hippocampal and amygdala volumes, corrected for total intracranial volume. In univariate tests secure versus insecure attachment group hippocampal volumes were not significantly different for either right (insecure *M *=* *4,065 mm^3^, *SD *= 468; secure *M *=* *3,925, *SD *= 385; *F*_1,55 _*= *0.77, *p *=* *.39) or left hemisphere (insecure *M *=* *4,059 mm^3^, *SD *= 509; secure *M *=* *3,885, *SD *= 376; *F*_1,55_*=*0.77, *p *=* *.39). For amygdala volumes univariate tests indicated significant group differences for right (insecure *M *=* *1,544 mm^3^, *SD *= 288; secure *M *=* *1,362, *SD *= 266; *F*_1,55_*=*5.07, *p *=* *.028), but not left hemisphere (insecure *M *=* *1,389 mm^3^, *SD *= 175; secure *M *=* *1,327, *SD *= 204; *F*_1,55 _*= *0.77, *p *=* *.39)

Infant attachment status was investigated as a predictor of hippocampal and amygdala volumes. First, we completed a 2 × 2 ANCOVA examining hippocampal volume in relation to infant attachment status (secure/insecure) and laterality (left vs. right hemisphere), with TIV, gender, participant history of depression and maternal total months of depression included as covariates. Results are depicted in Figure1. We found no significant effects of attachment on hippocampal volume, either as a main effect (*F*_*1,52 *_*= *0.12, *p *=* *.73, partial *η*^2 ^= .002) or in interaction with hemisphere (*F*_*1,52 *_*= *0.04, *p *=* *.85, partial *η*^2^ = .001).

Second, an equivalent analysis was completed for amygdala volume. Results indicated a significant main effect of infant attachment (*F*_*1,55 *_*= *4.39, *p *=* *.041, partial *η*^2 ^= .074). As can be seen in Figure1, in line with hypotheses, greater amygdala volumes were found in adults who had been insecurely attached infants relative to their securely attached counterparts. There was no significant attachment x laterality interaction (*F*_*1,55 *_*= *2.54, *p *=* *.12, partial *η*^2 ^= .044). Given that the presence/absence of maternal PND was a key defining feature of the sample which was strongly related to attachment, we repeated analyses including PND status as a second independent variable. The attachment main effect was retained (*F*_1,53 _= 4.66, *p *=* *0.035, partial *η*^*2 *^= .081) and no significant effect of maternal PND emerged (*F*_1,53 _= 0.41, *p *=* *0.53, partial *η*^*2 *^= .008). Importantly, the maternal PND by attachment status interaction term was also not significant and the estimated effect size was extremely small (*F*_1,53 _< 0.001, *p *=* *.99, partial *η*^*2 *^< .001). Thus, we had no reason to believe that associations between attachment status and amygdala volume were particularly present in one or other of the original recruitment groups.

## Discussion

In our sample of young adults who had been studied from birth, we found that the quality of the early mother–child relationship was a significant predictor of amygdala volume in adulthood. Specifically, participants who had insecure attachments at 18 months of age had larger amygdala volumes at 22 years than those who had been securely attached. By contrast, we did not find the quality of the early attachment relationship to be a predictor of hippocampal volumes.

Our finding that insecure attachment in infancy predicts amygdala volume in adulthood is consistent with previous reports of enlarged amygdalae, but no changes in hippocampi, in children who experienced early institutional deprivation (Mehta et al., 2009; Tottenham et al., 2010). The majority of insecure attachments (87%) in the current sample were Type A-Avoidant, which is typical of dyads in which mothers are relatively unresponsive to their infant (Ainsworth et al., 1978). As such, our observations complement the conclusions from the postinstitutional adoption studies, by suggesting that normative variations in the extent to which the infant experiences an involved and responsive caregiver may also predict amygdala morphology. With regard to the underlying mechanisms, in principle differences in amygdala volumes may arise as a consequence of more frequent or prolonged activation of the developing child's stress response systems. Not only is attachment conceptualized as both a consequence of less optimal parenting and a behavioural mechanism for regulating resultant stress, but several studies have also found direct evidence that insecurely attached infants show greater stress reactivity, as indicated by cortisol response to separation and other stressors (Fearon, Groh, Bakermans-Kranenburg, van IJzendoorn, & Roisman, 2014; Fox & Hane, 2008). Research from the animal literature demonstrates that early stress can significantly influence amygdala development (Lupien et al., 2009). Based on the current observations, the possibility that similar influences operate in human development warrants further investigation. Nevertheless, other mechanisms should also be considered, particularly given the correlational nature of our study. Greater amygdala volumes may be related to persistent environmental or behavioural effects occurring in association with insecure infant attachment status, may be a contributor to versus a consequence of attachment status, or may reflect underlying, shared genetic influences.

The functional implications of the current findings remain to be determined. Theoretically, attachment insecurity is held to represent a risk factor for a number of adverse outcomes, particularly in the socioemotional domain (Cassidy & Shaver, 2008). Previous observations from the same sample studied here have found mother–infant attachment patterns to be an important predictor of offspring socioemotional adjustment and responding in childhood and adolescence (Murray et al., 2006, 2011). As already described, insecure attachments are also associated with greater physiological reactivity to stress, which is likely to be directly influenced by neural activity in the amygdala (Fox & Hane, 2008; Whalen & Phelps, 2010). Previous research has indicated that greater amygdala volumes are associated with trait negative affectivity (Holmes et al., 2012), sensitivity to negative experiences (Barros-Loscertales et al., 2006; Gerritsen et al., 2012), and with the presence of elevated levels of anxiety in both children and adults (Baur et al., 2012; MacMillan et al., 2003; Qin et al., 2014), including in the aforementioned study of postinstitutionalized children (Tottenham et al., 2010). Such findings are notable, given that meta-analysis of attachment-related outcomes has indicated that avoidant attachments, in particular, are associated with elevated rates of internalizing symptoms (Groh, Roisman, van IJzendoorn, Bakermans-Kranenburg, & Fearon, 2012), and suggest that the larger amygdalae observed in the insecure group in the current study may predispose to elevated sensitivity to stress and/or symptoms of anxiety. Nonetheless, we found no association between participant lifetime history of anxiety or depressive disorder and amygdala volumes in our sample. Moreover, in an fMRI study of the same sample studied here, we also found no differences in amygdala responding during an emotion regulation task between those who were securely versus insecurely attached during infancy (Moutsiana et al., 2014). These observations are certainly not conclusive; the fMRI task utilized in our previous report focused on effortful regulation of emotion and particularly recruited prefrontal versus limbic neural regions; and we have limited power to detect associations between amygdala volumes and the presence of psychiatric disorder. However, direct evidence to show that the volumetric differences in amygdalae we observed in association with infant attachment security are of functional importance is still needed.

We failed to confirm previous reports of links between hippocampal volume and aspects of the caregiving environment, although we note that previous findings have been mixed. Thus, while one study found that maternal support in early childhood predicted larger hippocampal volumes in school-aged children (Luby et al., 2012), a second study found that higher levels of maternal warmth in childhood predicted smaller hippocampal volumes in adolescence (Rao, Betancourt et al., 2010). Such discrepancies are likely to reflect differences in the ages of the samples and the types of developmental experiences studied. It is worth noting that we did find that history of offspring and maternal depression were both related to greater hippocampal volume. However, these findings were in the opposite direction to that reported in some previous research, where smaller hippocampal volumes have been linked to a familial history of depression (Baare et al., 2010; Chen et al., 2010; Rao, Chen, et al., 2010). Given that other studies have also failed to find any association between familial risk of depressive disorder and hippocampal volumes (Luby et al., 2012; Lupien et al., 2011), or have identified larger hippocampi in association with risk status (van Erp et al., 2012; Romanczuk-Seiferth et al., 2014; Ladouceur et al., 2008), the current evidence base does not allow for clear conclusions.

The current study has some key strengths, including the longitudinal design and the rigorous observational assessment of attachment status. There are also some limitations. We have applied overall volumetric measurement, which is a gross indicator of neural morphology that potentially misses fine-grained structural differences that might be detectable in larger samples, or with more sophisticated (e.g. multimodal) or higher field strength scanning protocols. Moreover, findings are essentially correlational and causal influences cannot be inferred. Finally, there are some limitations in our ability to generalize findings; insecure attachments were almost exclusively of the avoidant type and studies of insecure ambivalent or disorganized attachment groups may yield different findings; and the sample had some unique characteristics, including the low risk profile, and the inclusion of a group with maternal PND. Nonetheless, the current analyses provide a proof of principle, demonstrating that the caregiving experiences captured by early attachment status can predict amygdala structure in adulthood. It remains for future studies to evaluate the significance of our observations and to explore the environmental and/or genetic factors that may have contributed to them.

**Key points**

- Animal research indicates that the quality of care received early in life influences morphological development in neural structures related to emotional processing.
- In humans, infant attachment status is considered a key indicator of early parenting.
- In a longitudinal study, we examined infant attachment as a predictor of hippocampal and amygdala volumes in early adulthood.
- Insecure versus secure attachment status in infancy predicted larger amygdala volume in adulthood, but was unrelated to hippocampal volume.
- We conclude that normative variations in the quality of early experience are relevant to amygdala morphological development, with possible implications for emotion processing and adjustment.
